# Graphene/Metal Composites Decorated with Ni Nanoclusters: Mechanical Properties

**DOI:** 10.3390/ma17235753

**Published:** 2024-11-24

**Authors:** Vyacheslav Kolesnikov, Roman Mironov, Julia Baimova

**Affiliations:** 1 Ufa Physical-Technical Institute, University of Science and Technology, Z. Validi 32, 450076 Ufa, Russia; slavakolesnikov102@gmail.com (V.K.); mayrxd@mail.ru (R.M.); 2 Institute for Metals Superplasticity Problems of the Russian Academy of Sciences, 450001 Ufa, Russia; 3 World-Class Research Center for Advanced Digital Technologies, Peter the Great St. Petersburg Polytechnic University, 195251 St. Petersburg, Russia

**Keywords:** composite, graphene, molecular dynamics, mechanical properties, strength

## Abstract

With the developments in nanotechnology, the elaborate regulation of microstructure shows attractive potential in the design of new composite materials. Herein, composite materials composed of graphene network filled with metal nanoparticles are analyzed to optimize the fabrication process and mechanical properties. In the present work, molecular dynamic simulations are used to analyze the possibility of obtaining a composite structure with Ni-decorated graphene. The weak bonding at the graphene–copper and graphene–aluminum interfaces is manipulated by functionalizing graphene with nickel nanoclusters. It is found that Ni decoration considerably increases interfacial bonding and, at the same time, prevents the formation of a strong graphene network. It is found that Ni decoration for the Al/graphene composite increases the its ductility by 0.6, while increasing it for the Cu/graphene composite by about 0.5. Ultimate tensile strength of the composite with Al and Cu is close and equal to 22 GPa, respectively. The strength of the composite with Ni-decorated graphene is much lower and equal to 13 GPa for Cu/graphene/Ni and 17 GPa for Al/graphene/Ni. While Young’s modulus for the Cu/graphene composite is 18 GPA, for Al/graphene, Al/graphene/Ni, and Cu/graphene/Ni, it is 12 GPa. The obtained results demonstrate the future prospects of the graphene modification for better composite enhancement.

## 1. Introduction

There is a rapidly growing demand for the design and development of new high-performance materials that offer superior properties to conventional materials. Composite materials are found to be at the forefront of technological innovation because they can exhibit properties that are much better than those of their own components. Graphene and carbon nanotubes are promising reinforcement candidates due to their unique mechanical properties [1,2,3]. Novel graphene-based composites demonstrate an outstanding combination of properties, including high specific strength and stiffness, high thermal conductivity, and enhanced resistance to environmental degradation [4,5,6,7,8,9,10,11,12]. Although a great deal of research has been carried out to date, we still have a long way to go before we gain an understanding of their properties and morphology control. Numerous problems arise during the fabrication process: low interface strength between graphene and metal, inhomogeneous distribution of the graphene reinforcement in the matrix, and the appearance of defects or other chemical additions during synthesis.

One of the well-known and very effective materials for engineering is Al, which is used in numerous applications because of its lightness, resistance to environmental factors, high thermal and electrical conductivity. Its main disadvantage is low strength [13,14]. High-strength aluminum materials are required for aerospace engineering and automotive products to meet extreme working environments [15,16,17]. Numerous works are devoted to the development of new promising graphene/Al composites [18,19,20,21,22]. One of the fundamental challenges is the difference in the bonding characteristics between aluminum and graphene, which are connected by weak van der Waals bonding. Moreover, the bonding energy between graphene and Al is very low, which prevents the formation of a strong composite structure.

A very similar situation exists with copper (Cu), which also has excellent properties: high strength, corrosion resistance, and thermal conductivity. However, graphene reinforcement can considerably increase not only Cu’s strength but also modify other important characteristics [23]. Graphene/Cu composites are considered as promising structural/functional materials for future applications [11,24,25,26]. Again, one of the main problems we face is improving the interfacial bonding between the metal matrix and the reinforcement.

The wettability of graphene with metal needs to be improved to enhance interfacial bond strength [27]. It has been shown that graphene/metal interactions can depend on the very different factors: defects, graphene curvature, and the presence of other atoms [28]. Different methods have been developed to date to increase the interfacial adhesion energy, such as molecular-level mixing processes and spark plasma sintering processes [24]. Graphene functionalization has also been effectively used due to its remarkable and tunable physicochemical properties [29,30]. One of the possibilities to increase the strength of the graphene/metal interface is graphene decoration via metal atoms. For example, nickel (Ni) strongly interacts with graphene flakes, can be easily anchored to graphene, and affects the resulting properties [31,32,33].

Previously, graphene decoration with Ni has been widely used for various purposes, such as hydrogen storage, to enhance the strength of graphene-reinforced composites or conductivity performance [34,35,36,37,38]. It was found that Ni-coated graphene exhibited a significantly enhanced strengthening effect compared to pure graphene reinforcements of aluminum matrix composites [35]. It was found that as the content of Ni-coated graphene increased from 0 to 1.5 wt%, Vickers hardness, strength, and Young’s modulus of the composites increased continuously due to the good dispersion of Ni-coated graphene in the Al matrix. The strength and ductility of Al composites reinforced by Ni-coated graphene depend on the time of Ni deposition [36]. Therefore, the Ni decoration method has been widely used to improve the composite properties; however, numerous problems have been raised. For example, this method has mainly been used for metal matrices filled with graphene, but not for graphene foams filled with metal nanoparticles, which is a more novel composite architecture. Regarding pure graphene, its main strengthening mechanism is dislocation strengthening. In addition, the resulting strength of the composite is difficult to control as it is strongly dependent on the distribution of Ni-coated graphene and Ni content.

The results show that Ni is the most powerful coating metal among Ni, Cu, and Ti in enhancing the interfacial strength of Al/graphene composites [39,40] and Cu/graphene composites [41]; however, this has also been found for metal matrix composites. The new structural form of the composite, which has recently become very popular due to its high strength, ductility and conductivity, is the graphene network combined with metal [42,43,44,45,46]. It has been shown that discrete graphene flakes (GFs) coated on Ni particles are assembled into an intact and continuous graphene network through interlayer and lateral connections. Moreover, the formation of a strong graphene network was observed for a graphene-like metal such as Ni, while the results for Cu and Al were much worse [43,47]. The poor bonding between Al (Cu) and GFs results in the formation of a graphene network filled with large metal nanoparticles, which consequently become fracture centers. Significant progress has been made in the synthesis and characterization of such composites, and understanding of property control and structure modification is important for their future performance [48,49,50].

Molecular dynamics (MD) simulation can be a very helpful tool for searching new material, as well as for a detailed analysis of their properties and performance [51,52,53,54]. The review works of [51,52] showed how atomic-scale simulations can be used to investigate the microscopic structure–property relationships of nanocarbon-reinforced metal matrix composites, in which the physical properties, crystal orientations, interfacial structures, and mechanical behaviors could be studied under a wide range of working temperatures and strain rates. The tensile properties of different composites have been extensively studied via MD simulations [43,47,54,55,56]. The existing potentials are very effective for the description of new carbon and new composite materials, which allow for their successful applications [47,57]. The series of works by [43,47,52,55,56] studied different aspects of MD simulations concerning Me/graphene composites (graphene networks filled with Me nanoparticles) from the choice of interatomic potentials [47,52] to the detailed analysis of their physical and mechanical properties. It was found that the main factors affecting the fabrication of a composite are the sizes of nanoparticles, the orientations of structural units, and the temperatures of hydrostatic compression [55]. It was found that the higher the Ni content in the composite, the higher the thermal conductivity; however, this decreases the composite strength [56]. Based on the obtained results, new important issues have been revealed: the need to increase the bonding between GF and metal, as well as the need to analyze their property control to obtain composites with specific properties.

In the present work, the modification of a graphene surface with Ni nanoclusters for Al/graphene and Cu/graphene composites is analyzed by molecular dynamic simulations. The mechanical strength of these Al/graphene and Cu/graphene composites is investigated for modified and unmodified structures with a full description of all structural changes.

## 2. Simulation Details

All simulations are performed using MD, which allows fpr the mathematical description of interatomic interactions. Classical methods of interaction are described by the potential function $U({\vec{r}}_{1},{\vec{r}}_{2},\ldots ,{\vec{r}}_{N})$, which determines the potential energy of a system as a function of their coordinates. For the model system consisting of *N* particles, Newton’s equations of motion are solved until the properties of the system no longer change with time. The forces acting on each atom are calculated as follows:

$${\vec{F}}_{i}=-\frac{\partial U({\vec{r}}_{1},{\vec{r}}_{2},\ldots ,{\vec{r}}_{N})}{\partial {\vec{r}}_{i}}\equiv -\nabla_{i}U\left({\vec{r}}_{1},{\vec{r}}_{2},\ldots ,{\vec{r}}_{N}\right).$$

At the approximation of pair potentials, the energy of a system of particles is represented as the sum of potential energy interactions of all pairs of atoms:

$$U\left({\vec{r}}_{1},{\vec{r}}_{2},\ldots ,{\vec{r}}_{N}\right)=\frac{1}{2}\sum_{i=1}^{N}\sum_{j=1,(j\neq i)}^{N}\varphi \left(r_{i,j}\right),$$

where $r_{i,j}=\left|r_{j}-r_{i}\right|$ is the distance between a pair of atoms. The most common pair potentials are the Lenard–Jones and Morse potentials.

The interatomic interaction in this type of complex system can be described with the following hybrid potential function:

$$U=U_{Me-Me}+U_{C-C}+U_{Me-C}+U_{Me-Ni}+U_{C-Ni}+U_{Ni+Ni},$$

where $U_{Me-Me}$ describes the interaction between metal nanoparticles, $U_{C-C}$ describes the covalent interaction within the graphene network, $U_{C-Me}$ describes the van der Waals interaction between the graphene surface and metal nanoparticles, $U_{Me-Ni}$ ($U_{C-Ni}$) describes the interaction between the main metal in the composite (graphene network) and Ni nanoclusters, and $U_{Ni+Ni}$ describes the interaction between Ni nanoclusters.

The interaction between carbon atoms is described using the well-known AIREBO potential [58]. Previously, this potential has been successfully used to study the formation of pure carbon nanoparticles [59], the deformation behavior of the Me/GF composites based on graphene networks [43,52] and zero misorientation interfaces in graphene [60], for example.

(1) $$U_{C-C}=\frac{1}{2}\sum_{i}\sum_{i\neq j}\left[U_{ij}^{REBO}+U_{ij}^{LJ}+\sum_{k\neq i,j}\sum_{l\neq i,j,k}U_{kijl}^{TORSION}\right],$$

where $U_{ij}^{REBO}$ is the hydrocarbon REBO potential, the $U_{ij}^{LJ}$ term adds longer-ranged interactions using a form similar to the standard LJ potential, and $U_{kijl}^{TORSION}$ describes various dihedral angle preferences in hydrocarbon configurations. This potential is very famous for the study of different carbon structures and their properties.

The Ni-C or Al-C interaction is described by the Morse interatomic potential, which can be successfully applied to reproduce van der Waals interactions between graphene and metal. Previously, Me/graphene composites with different architectures have been investigated using Morse or Lennard–Jones (LJ) potentials [26,45,61].

The Lennard–Jones (LJ) potential can be described as

$$U\left(r_{ij}\right)=4\epsilon \left[{\left(\frac{\sigma }{r_{ij}}\right)}^{12}-{\left(\frac{\sigma }{r_{ij}}\right)}^{6}\right],$$

where α and ϵ are constants having dimensions of length and energy, respectively.

Morse potential can be written as

(2) $$U^{Ni-C}\left(r\right)=D_{e}\left[{\left(1-e^{-\alpha (r-R_{e})}\right)}^{2}-1\right],$$

where $D_{e}$ is the binding energy, $R_{e}$ is the distance for the minimum potential energy, and 1/α describes the width of the potential.

Parameters for all the applied potential functions are presented in Table 1.

**Figure 1:**
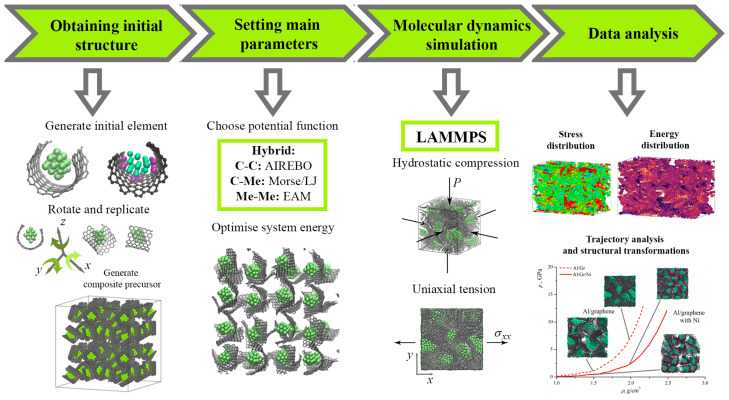
Molecular dynamic simulation chart for the analysis of the mechanical properties of the Me/graphene composite.

Figure 1 presents the MD simulation chart for the analysis of the mechanical properties of the Me/graphene system.

**Figure 2:**
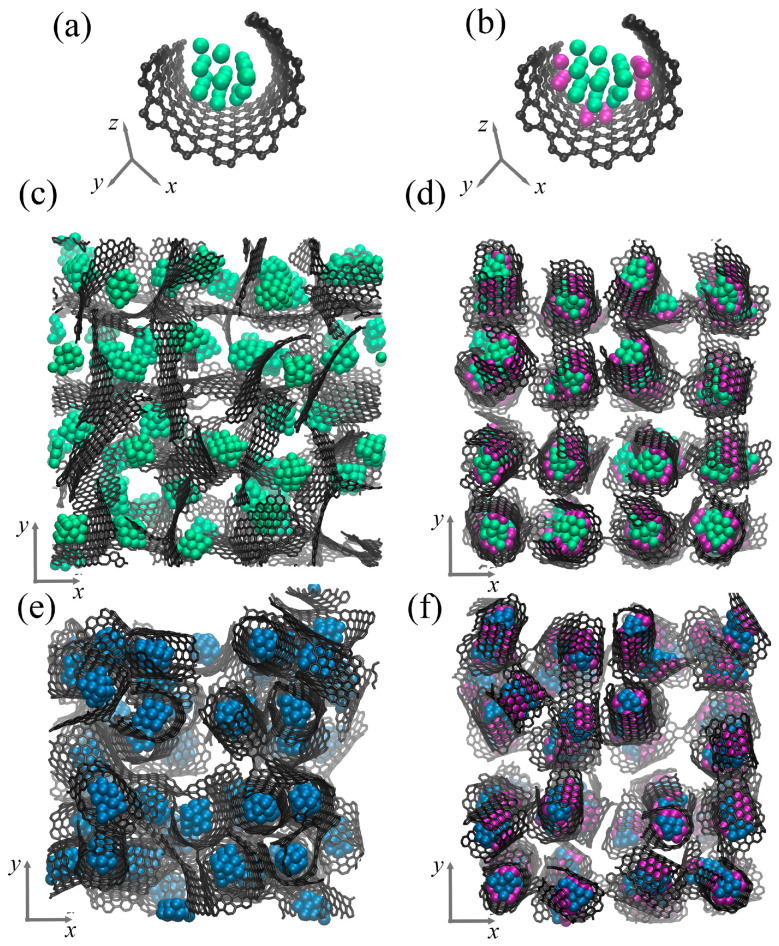
Single structural element without (**a**) and with (**b**) Ni nanoclusters. (**c**,**d**) Initial composite precursor for Al/GF and Al/GF/Ni. (**e**,**f**) Initial composite precursor for Cu/GF and Cu/GF/Ni. Graphene is shown by grey color, Al atoms—by green color, Ni atoms—by purple color, Cu atoms—by blue color.

Figure 2 presents the structural element of the composite in a perspective view and the initial composite precursor in accordance to the xy-plane. As can be seen, the structural element consists of a graphene flake (GF) and metal (Me) nanoparticle inside. The graphene flake is decorated by the Ni nanoclusters (shown in purple). This structural element is randomly rotated and repeated four times along all axes to obtain the composite precursor. For simplicity, the composites are named as Al/GF and Cu/GF; Al/GF/Ni and Gu/GF/Ni for Ni-decorated structures. The final structural state of the composite precursor after relaxation is shown in Figure 2c,d for the Al/GF system and in (e,f) for the Cu/GF system. After relaxation, the initial ideal shape of the GF is changed according to the interatomic potential function acting on the system.

To obtain the composite, hydrostatic compression (HC) is applied at room temperature for Al/GF and Al/GF/Ni composites. For Cu/GF and Cu/GF/Ni composites, HC is applied at two temperatures—300 and 600 K. Compression is applied to the composite precursor after relaxation (Figure 2c,d), which was previously described in detail in [43,47,55,56]. It has been found that better mechanical properties can be obtained for the composite obtained at $T_{HC}=0.7T_{m}^{NP}$ [43], where $T_{m}^{NP}$ is the melting temperature of the nanoparticle. Therefore, here, the temperature of the deformation treatment is chosen according to the melting temperature of the metal nanoparticles: $T_{m}^{Cu}$ = 720 K [68] and $T_{m}^{Al}$ = 488 K [69]. The HC is performed with a strain rate of 0.01 ps^−1^ at 300 K for the Al/GF composite; for the Cu/GF composite, the treatment temperatures are 300 K and 600 K with the same strain rate. The two temperatures are chosen to analyze how the decoration of GFs with Ni nanoclusters affects the required HC temperature.

First, the obtained composites are equilibrated with the NPT ensemble at 300 K. Then, uniaxial tensile stress at 300 K is applied to the composites along the *x*-axes, and the corresponding stress components are calculated. The strain rate is $\dot{\varepsilon }$ = 0.005 ps^−1^.

All the simulations are performed in the LAMMPS simulation package [70,71,72]. The equations of motion for the atoms are integrated using the fourth-order Verlet method with a time step of 0.2 fs. Periodic boundary conditions are applied along the *x*-, *y*-, and *z*-axes. The Nose–Hoover thermostat is used to control the system temperature when simulating the relaxation and tension. The molecular graphics programs VMD (Visual Molecular Dynamics) and OVITO are used to visualize the structure [73,74].

## 3. Results and Discussion

### 3.1. Hydrostatic Compression

**Figure 3:**
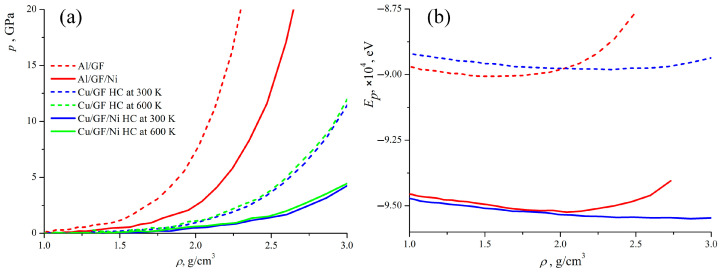
Pressure (**a**) and potential energy (**b**) as the function of composite precursors’ density during hydrostatic compression. Pressure–density curves are presented for all the considered structures. Energy–density curves are presented just for four representative structures Al/GF, Al/GF/Ni, Cu/GF, and Cu/GF/Ni.

Figure 3a presents the pressure–density curves obtained during the compression of the composite precursor. Two curves are compared for Al/GF and Al/GF that are decorated with Ni; two curves for Cu/GF with and without Ni for HC at 300 K; and two curves for HC at 600 K. It is observed that the pressure is zero when ρ < 1 g/cm^−3^. This occurs because the structural units are far away from each other and start to interact only at ρ = 1 g/cm^−3^. Also, in this case, it is better to consider the density of the structure rather than the strain. This can better characterize the composite formation or the transition from the precursor to the composite. Until the empty spaces between the GFs are removed, the real deformation of the structure cannot be considered. In all cases, the rigidity of the composite precursor increases with its density, due to the formation of new van der Waals and covalent bonds between neighboring GFs and new covalent bonds at the edges (more details on structure transformation are given below). This behavior is explained by the special mechanical response of the graphene network to compression.

Previously, it was shown that better mechanical properties for Cu/GF composites would be achieved after HC at 600 K [43,47,55,56]; however, Ni decoration can result in a decrease in the temperature required for fabrication. Therefore, the treatment temperature is also analyzed for the fabrication of Cu/GF composites with increased strength.

**Figure 4:**
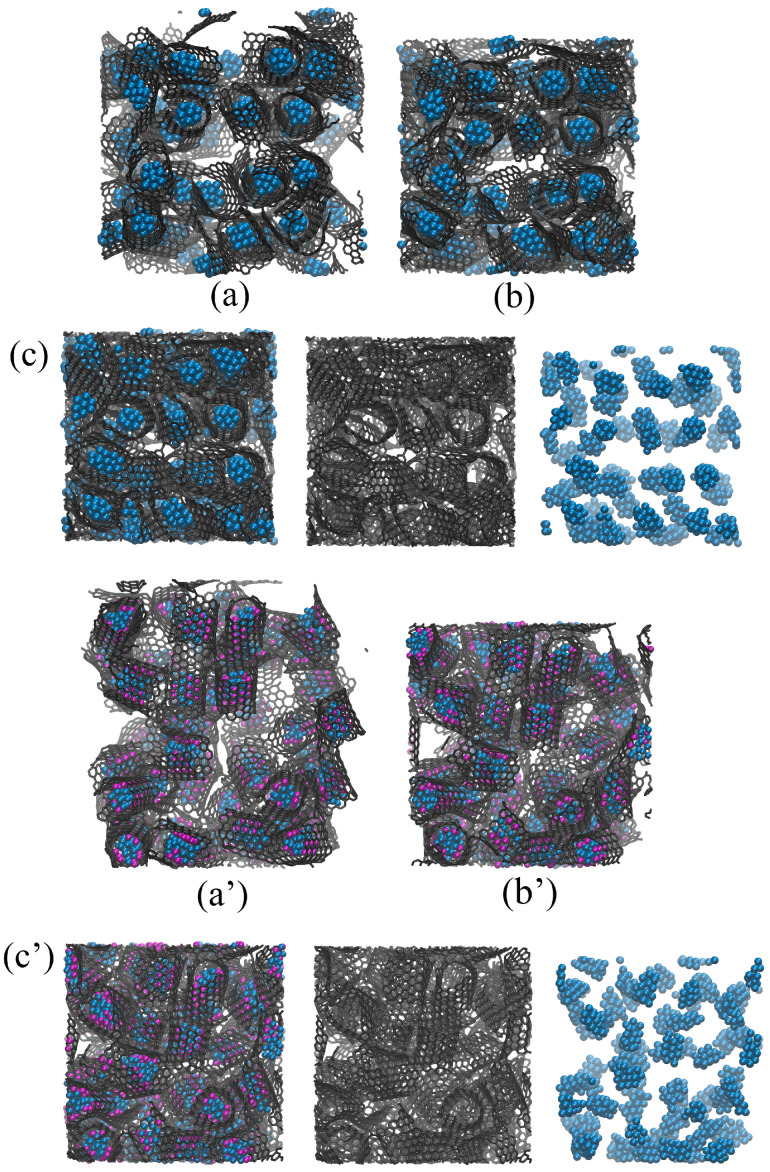
Snapshots of the Cu/GF composite precursor during HC at (**a**,**a’**) ρ = 1 g/cm^−3^, (**b**,**b’**) ρ = 2.0 g/cm^−3^, and (**c**,**c’**) ρ = 3.0 g/cm^−3^. In (**c**,**c’**), the composite is presented in three states: all atoms; only carbon atoms; and only Cu atoms. (**a**–**c**) Composite without Ni decoration and (**a’**–**c’**) composite with Ni nanoclusters. Carbon is shown in gray, Cu in blue, and Ni in purple.

As can be seen (green and blue curves in Figure 3a) for the Cu/GF composite, compression is performed in the same way at 300 and 600 K, with the pressure–strain curves being almost identical; however, the resulting strength of the composite can be very different. To better analyze the fabrication process, let us consider the structural transformations during compression. Figure 4 presents the snapshots of the Cu/GF and Cu/GF/Ni structures during HC at 300 and 600 K.

Figure 3b presents the potential energy $E_{p}$ of the composite precursors as a function of their density during compression. As can be seen, for all composites, the state with the minimum potential energy can be found, where the density of the structure is optimal. For Al/GF, equilibrium density is 1.4–1.7 g/cm^3^ with the minimum $E_{p}\left(min\right)$ = −9.0 eV; for Al/GF/Ni, the equilibrium density is 1.7–1.9 g/cm^3^ with the minimum $E_{p}\left(min\right)$ = −9.52 eV; for Cu/GF, the equilibrium density is 1.9–2.3 g/cm^3^ with the minimum $E_{p}\left(min\right)$ = −8.98 eV; and for Cu/GF/Ni, the equilibrium density is 2.3–2.7 g/cm^3^ with the minimum $E_{p}\left(min\right)$ = −9.55 eV. These differences in equilibrium densities of the composite precursors correlate with the differences in pressure–density curves for different structures. As can be seen, the energy of the structure with Ni is lower than for the Me/GF structure, which can be explained due to better bonding occurring in the presence of Ni atoms. The potential energy for the structures with Al increases much faster than for the structures with Cu, which supports the conclusion regarding the higher rigidity of structures with Al.

Figure 4 presents the snapshots of Cu/GF (a–c) and Cu/GF/Ni (a’–c’) structures during HC at 300 K as an example. For HC at 600 K, no differences were found except for the formation of a higher number of covalent bonds between graphene flakes. Thus, all the processes during compression can be analyzed from the snapshots for HC at 300 K. As can be seen, even after initial relaxation, composite precursors are very similar (see Figure 4). The copper nanoparticles are covered with graphene in both cases; however, in the presence of Ni clusters, the graphene “balls” are even denser, and the Cu atoms are strongly attached to the Ni/GF surface (which is found from the analysis of energy per atom). In the structure without Ni, Cu nanoparticles can coagulate when flake edges are opened. For Cu/GF/Ni, this coagulation of metal atoms is impossible. The distribution of nanoparticles over the graphene network is very similar in both cases.

**Figure 5:**
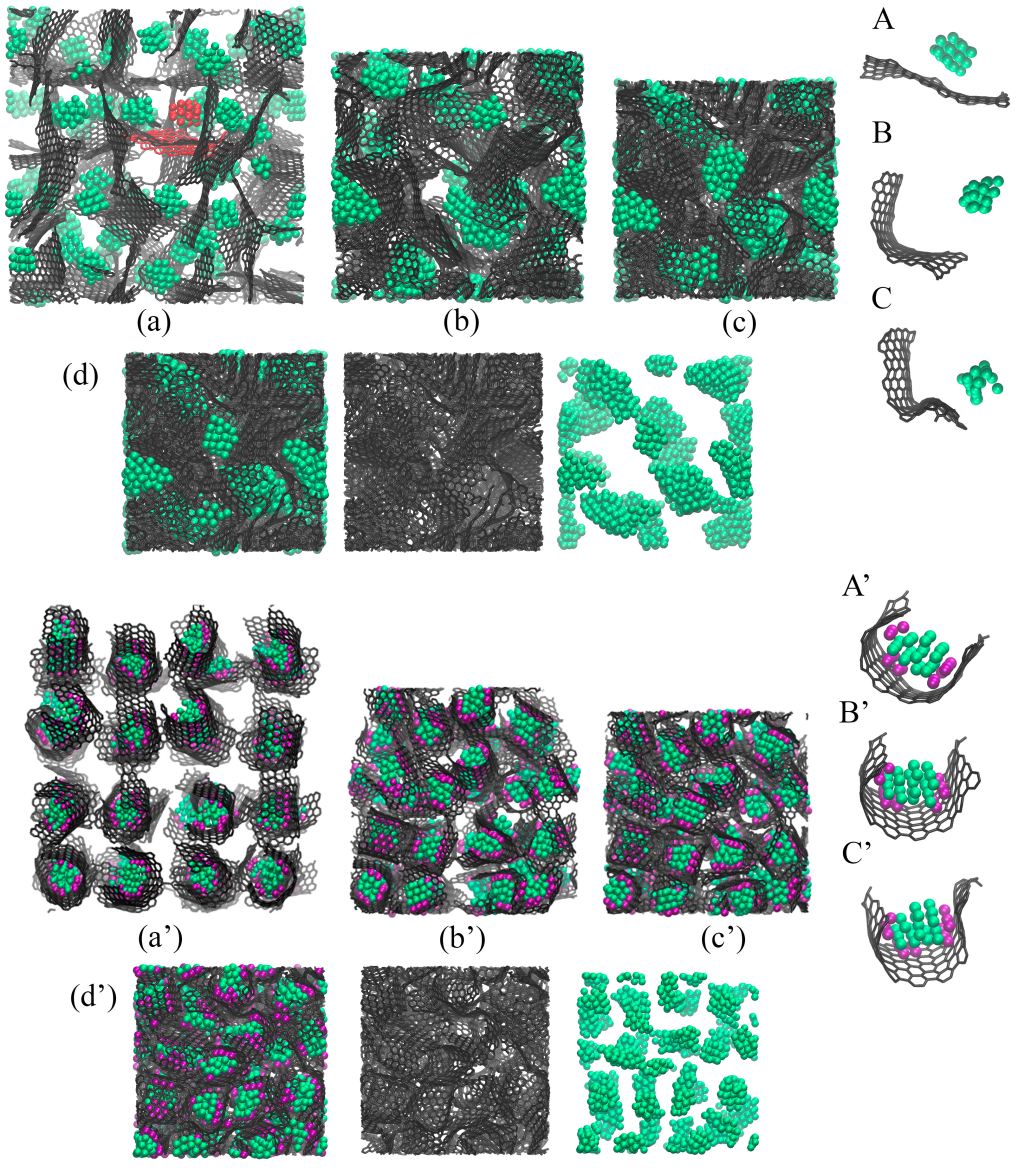
Snapshots of the Al/GF composite precursor during HC at (**a**,**a’**) ρ = 1 g/cm^−3^, (**b**,**b’**) ρ = 1.5 g/cm^−3^, (**c**,**c’**) ρ = 1.7 g/cm^−3^ and (**d**,**d’**) ρ = 2 g/cm^−3^. In (**d**,**d’**), the composite is presented in three states: all atoms; only carbon atoms; and only Al atoms. Snapshots of the single structural element during HC at (**A**,**A’**) ρ = 1 g/cm^−3^, (**B**,**B’**) ρ = 1.5 g/cm^−3^, (**C**,**C’**) ρ = 1.7 g/cm^−3^. (**a**–**d**) Composite without Ni decoration and (**a’**–**d’**) composite with Ni nanoclusters. Carbon is shown in gray, Al in green, and Ni in purple.

Figure 5 shows the snapshots of Al/GF (a–d) and Al/GF/Ni (a’–d’) structures during HC at 300 K. As can be seen, the composite precursors are very different even after initial relaxation. The presence of Ni decoration results in a significant anchoring of Al nanoparticles to the Ni/GF interface (see A’–C’; the structural element is the Al nanoparticle with Ni nanoclusters covered by graphene). This is very similar to the behavior for graphene, which completely covers Ni nanoparticles [43,47,55,56]; however, Al nanoparticles can be easily repelled by graphene. From the snapshots of individual flakes, it can be seen that free Al nanoparticles move away from graphene quickly. Moreover, it is clear that nanoparticles come closer to graphene only at a high compression level.

As can be seen from Figure 5a, without Ni decoration, GFs are freely moving, repelled by Al nanoparticles and are flattened, while Al tends to coagulate. In the structure with Ni, GFs are curved, strongly anchored with metal nanoparticles, and monotonously distributed inside the graphene network (see Figure 5b). From one point of view, this leads to an increase in the interfacial strength between Al and GF. From another point of view, this can prevent the formation of strong covalent networks because the edges of GFs are not as free.

As a result of the Ni decoration, we have two different structural morphologies. For Al/GF, it is a graphene network with a large surface area, filled with large Al nanoparticles, many bonds between neighboring flakes, and an increased number of $sp^{3}$-hybridizations. The inhomogeneous distribution of Al nanoparticles can be seen in Figure 5d. For Al/GF/Ni, metal nanoparticles are covered with graphene flakes, the composite structure is much denser, the graphene network has a lower number of covalent bonds, and Al nanoparticles are monotonically distributed over GFs (see Figure 5d’).

For Al/GF/Ni (Cu/GF/Ni), Ni strongly interacts with graphene and Al (Cu) nanoparticles, resulting in the formation of graphene “balls”. Ni nanoclusters also prevent the coagulation of Al nanoclusters (Cu nanoclusters). The presence of Ni nanoclusters results in a different pressure increase for both cases, simply due to the presence of an additional Ni element. The rapid pressure increase for Al/GF (Al/GF/Ni) is explained by the strong repulsion of Al and graphene: even at high compression level, Al nanoparticles are placed at a distance of about 5–7 Å from graphene flakes. Higher pressure and temperature are required to further compress the structure. In contrast, Cu can be easily attached to graphene at a minimum distance of 2.2 Å. The Cu/GF structure is less rigid than Al/GF due to this significant difference in the nature of he interaction between graphene and metal.

### 3.2. Uniaxial Tension

**Figure 6:**
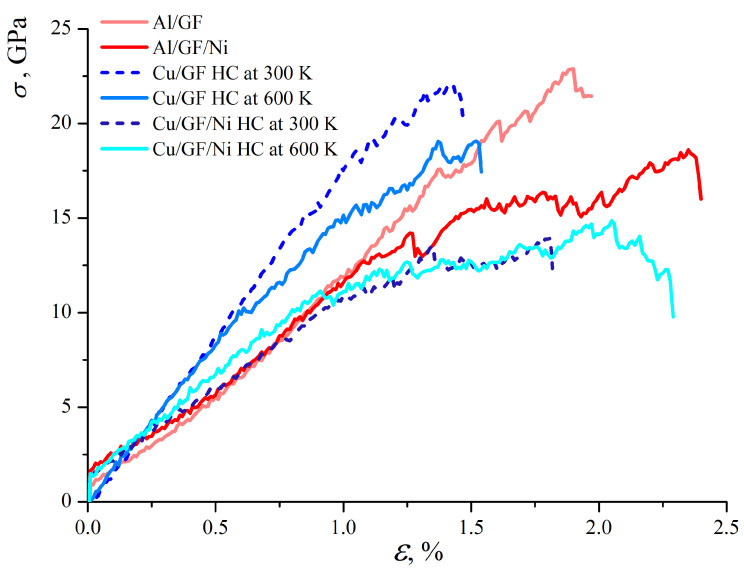
Stress–strain curves under uniaxial tension for all structures under consideration.

Figure 6 presents the stress–strain curves for composites under tension. Note that due to the difference in the interaction energies between graphene and Al (Cu), the tension is applied to Al/GF, which is compressed to ρ = 2.0 g/cm^3^, while Cu/GF is compressed to a higher density of ρ = 3.0 g/cm^3^. These values are chosen according to the stress per atom, energy state, and structural analysis. At ρ = 2.0 g/cm^3^, the Cu/GF structure still does not transform into a composite and is much weaker than the Al/GF composite.

As can be seen, the stress–strain curves for Cu/GF at 300 and 600 K are very close up to ε = 0.45. For higher strains, the difference is explained by the different structural changes: small oscillations in the stress–strain curve for Cu/GF obtained at 600 K are related to the continuous rearrangement of the carbon chains formed between the structural elements, with some covalent bonds breaking and new ones appearing during deformation. For the Cu/GF composite obtained at 300 K, this process starts only at ε = 1.0. For the Cu/GF composite obtained at 600 K, due to the thermal fluctuations during compression, a preferential formation of short chains occurs. These chains start to break during tensile stress at ε = 0.45. For the Cu/GF composite obtained at 300 K, more stable bonds are formed between flakes, resulting in higher strength. However, the Young’s modulus obtained from the initial part of the stress–strain curve is equal to 18 GPa.

**Figure 7:**
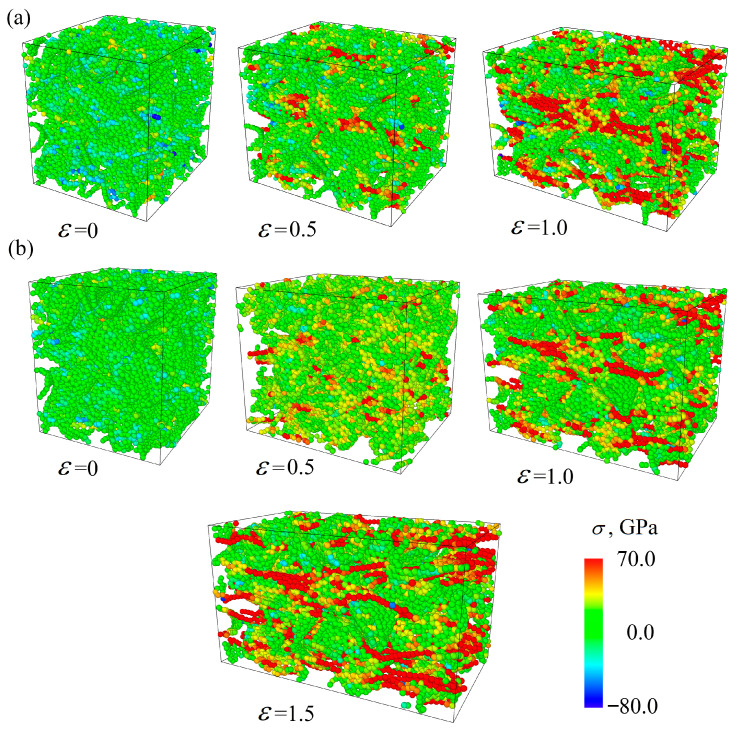
Stress per atom under tension for the Gu/GF (**a**) and Cu/GF/Ni (**b**) composites obtained by HC at 300 K.

Figure 7 presents the stress per atom for the Cu/GF composite (a) and for the Cu/GF/Ni composite obtained at HC 300 K as an example. Similar structural changes took place for the Cu/GF and Cu/GF/Ni composites obtained at HC 600 K. Here, the stress per atom is shown for only two cases as an example to better illustrate the process of composite deformation. The red-colored atoms are the straightest GFs., while the straightest carbon chains are seen at high strain levels. For example, at ε = 0.45, the number of such stressed sites in the structure is much lower for the Cu/GF composite obtained at 300 K than for the composite obtained at 600 K.

As can be seen from Figure 6, the strength of the Ni-decorated composite is much lower than that of the Cu/GF composite for both 300 and 600 K. The same is found for the Al/GF composite in comparison with the Al/GF/Ni composite. Young’s modulus for Cu/GF/Ni, Al/GF, and Al/GF/Ni is close and equal to 12 GPa.

For the Al/GF and Al/GF/Ni composites, the stress–strain curves are almost identical up to ε = 1.0. The distribution of stress per atom is very similar to that for Cu/GF and is not presented as a figure. Again, small drops in the stress–strain curve correspond to the rearrangement of the covalent bonds in the structure. Stressed GFs appeared at about ε = 0.6. Since Ni negatively affects the formation of graphene networks, structures without Ni are stronger.

Interestingly, the presence of Ni increases composite ductility but decreases its strength. It is found that Ni decoration for the Al/graphene composite increases composite ductility by 0.6. On the other hand, for Cu/graphene, it increases composite ductility by about 0.5. The structural analysis showed that without Ni, the obtained graphene network has better interconnection, as well as a larger number of atoms with $sp^{3}$-hybridization. The reason for adding Ni to the structure was to increase Me/GF’s interface strength for weakly interacting Al and Cu atoms. Analysis of the structure under compression showed that Ni definitely increases the interaction between Al/GF and Cu/GF, while simultaneously preventing the formation of a covalent graphene network. Thus, it is important to note that structural elements interact mainly with van der Waals bonds, resulting in a weakening of the composite.

## 4. Conclusions

A detailed comparative study was carried out using molecular dynamics to investigate the mechanical properties of Me/GF composites with modified structures. Nickel nanoclusters were chosen as graphene decorations.

It was found that during hydrostatic compression, the rigidity of the graphene network increased with compressive strain. The structure with Al was more rigid than Cu/GF due to the strong repulsion of Al and graphene. The fabrication temperature affected the resulting mechanical properties of the Cu/GF composite. The ultimate tensile strength was higher for the Cu/GF composite obtained at 300 K, due to better bonding between neighboring graphene flakes. Thermal fluctuations at 600 K resulted in a faster rearrangement of covalent bonds, causing a strong graphene network to appear. In contrast, with Ni decoration, the composite obtained at 600 K was more ductile. Moreover, thermal fluctuations positively affected the formation of new covalent bonds.

It was found that Ni decoration leads to a decrease in the strength of composites and causes an increase in their ductility. The interfacial interaction between Al (Cu) and GF was significantly increased, but it prevented the formation of a strong graphene network. It can be assumed that for layered metal matrix composites, the presence of Ni can actually increase final strength. However, for this particular morphology (graphene network filled with metal nanoparticles), Ni decoration negatively affected composite strength.

It was found that hydrostatic compression allows us to obtain Al/GF composites with increased strength and ductility. This is possible due to special composite morphology: Al nanoparticles which are normally strongly repelled by graphene are covered by graphene flakes at a high pressure and cannot move out of pores. For Al, this method of composite fabrication is preferable because layered structures can be easily destroyed due to the weak interaction between graphene and Al. The obtained results explain how to control the properties of Me/GF composites with temperature–pressure treatments and graphene functionalization.

## Figures and Tables

**Table 1:** Potential function parameters of Morse [62,63,64], LJ [65], EAM [66,67], and AIREBO [58]. Here, $D_{e}$ is the binding energy, 1/α describes the width of the potential, $R_{e}$ is the equilibrium distance of the two atoms for Morse potential; ϵ is the depth of the potential, σ is the distance at which the particle–particle potential energy is zero for the LJ potential, and $r_{c}$ is the cut-off radius for all the potentials used.

System	Parameter	Potential
Al-C	$D_{e}$ (eV)	0.196
	α (1/Å)	4.117
	$R_{e}$ (Å)	3.450
	$r_{c}$ (Å)	4.0
Ni-C	$D_{e}$ (eV)	0.433
	α (1/Å)	3.244
	$R_{e}$ (Å)	2.316
	$r_{c}$ (Å)	4.5
Cu-C	ϵ (eV)	0.019996
	σ (Å)	3.225
	$r_{c}$ (Å)	8.0625
C-C	$r_{c}$	3.0

## Data Availability

The data presented in this study are available on request from the corresponding author due to privacy restrictions.

## Abbreviations

The following abbreviations are used in this manuscript:

MD	Molecular dynamics
GF	Graphene flake
HC	Hydrostatic compression
Me	Metal

## References

[1] Ilgamov M.A., Aitbaeva A.A., Pavlov I.S., Dmitriev S.V. (2024). Carbon Nanotube Under Pulsed Pressure. Facta Universitatis, Series: Mechanical Engineering.

[2] Gao T., Huang H., Huang J., Chen Q., Xiao Q. (2024). Molecular dynamic studies of the micromechanical response of titanium–aluminum layered twin structures and graphene. Mech. Mater..

[3] Gao T., He H., Liu Y., Bian Z., Chen Q., Xie Q., Liang Y., Xiao Q. (2023). Molecular dynamics simulation of dislocation network formation and tensile properties of graphene/TiAl-layered composites. Surfaces Interfaces.

[4] Zhang X., Gao J., Zhang L., Chen Y., Zhang Y., Zhang K. (2024). Molecular Dynamics Study on the Sintering Mechanism and Tensile Properties of Novel Cu Nanoparticle/Graphene Nanoplatelet Composite Solder Paste. Materials.

[5] Yankovaskaya U.I., Korznikova E.A., Korpusova S.D., Zakharov P.V. (2023). Mechanical Properties of the Pt-CNT Composite under Uniaxial Deformation: Tension and Compression. Materials.

[6] Süzer İ., Hayirci S.B., Boyaci E., Deniz A., Mertdinç-Ülküseven S., Öveçoğlu M.L., Gökçe H., Ağaoğulları D. (2024). Graphene nanoplatelet reinforced Al-based composites prepared from recycled powders via mechanical alloying and pressureless sintering. Diam. Relat. Mater..

[7] Devarajan B., Lakshminarasimhan R., Murugan A., Rangappa S.M., Siengchin S., Marinkovic D. (2024). Recent Developments in Natural Fiber Hybrid Composites for Ballistic Applications: A Comprehensive Review of Mechanisms and Failure Criteria. Facta Universitatis, Series: Mechanical Engineering.

[8] Yu M., Yuan X., Guo J., Tang N., Ye S., Liang J., Jiang L. (2021). Selective graphene-like metal-free 2D nanomaterials and their composites for photocatalysis. Chemosphere.

[9] Zhao Z., Bai P., Du W., Liu B., Pan D., Das R., Liu C., Guo Z. (2020). An overview of graphene and its derivatives reinforced metal matrix composites: Preparation, properties and applications. Carbon.

[10] Kausar A., Ahmad I., Zhao T., Aldaghri O., Ibnaouf K.H., Eisa M.H. (2023). Graphene Nanocomposites as Innovative Materials for Energy Storage and Conversion—Design and Headways. Int. J. Mol. Sci..

[11] Kazakov A.M., Korznikova G.F., Tuvalev I.I., Izosimov A.A., Korznikova E.A. (2023). The Effect of Copper–Graphene Composite Architecture on Thermal Transport Efficiency. Materials.

[12] Doan D.Q., Vu H.C., Nguyen V.T., Vu T.Q., Tran V.T., Chu V.T. (2024). An atomic-scale insight into mechanical enhancement and frictional properties of amorphous/graphene multilayers. Tribol. Int..

[13] Li Q., Xue S., Wang J., Shao S., Kwong A.H., Giwa A., Fan Z., Liu Y., Qi Z., Ding J. (2018). High-Strength Nanotwinned Al Alloys with 9R Phase. Adv. Mater..

[14] Chen B.Q., Liu K., Xu S. (2024). Recent Advances in Aluminum Welding for Marine Structures. J. Mar. Sci. Eng..

[15] Williams J.C., Starke E.A. (2003). Progress in structural materials for aerospace systems11The Golden Jubilee Issue—Selected topics in Materials Science and Engineering: Past, Present and Future, edited by S. Suresh. Acta Mater..

[16] Pollock T.M. (2016). Alloy design for aircraft engines. Nat. Mater..

[17] Chen D., Li J., Sun K., Fan J. (2023). Graphene-reinforced metal matrix composites: Fabrication, properties, and challenges. Int. J. Adv. Manuf. Technol..

[18] Du Y., Wang M., Ye X., Liu B., Han L., Jafri S.H.M., Liu W., Zheng X., Ning Y., Li H. (2023). Advances in the Field of Graphene-Based Composites for Energy–Storage Applications. Crystals.

[19] Dadkhah M., Saboori A., Fino P. (2019). An Overview of the Recent Developments in Metal Matrix Nanocomposites Reinforced by Graphene. Materials.

[20] Saboori A., Dadkhah M., Fino P., Pavese M. (2018). An Overview of Metal Matrix Nanocomposites Reinforced with Graphene Nanoplatelets; Mechanical, Electrical and Thermophysical Properties. Metals.

[21] Zhou T., Lei M., Xu J. (2024). Recent progress in the development and properties of aluminum matrix composites reinforced with graphene: A review. Mater. Today Sustain..

[22] Aydın F. (2023). A review of recent developments in the corrosion performance of aluminium matrix composites. J. Alloys Compd..

[23] Cao M., Xiong D.B., Tan Z., Ji G., Amin-Ahmadi B., Guo Q., Fan G., Guo C., Li Z., Zhang D. (2017). Aligning graphene in bulk copper: Nacre-inspired nanolaminated architecture coupled with in-situ processing for enhanced mechanical properties and high electrical conductivity. Carbon.

[24] Hwang J., Yoon T., Jin S.H., Lee J., Kim T., Hong S.H., Jeon S. (2013). Enhanced Mechanical Properties of Graphene/Copper Nanocomposites Using a Molecular-Level Mixing Process. Adv. Mater..

[25] Hwang B., Kim W., Kim J., Lee S., Lim S., Kim S., Oh S.H., Ryu S., Han S.M. (2017). Role of Graphene in Reducing Fatigue Damage in Cu/Gr Nanolayered Composite. Nano Lett..

[26] Shuang F., Aifantis K.E. (2021). Dislocation-graphene interactions in Cu/graphene composites and the effect of boundary conditions: A molecular dynamics study. Carbon.

[27] Zhao Z.Y., Guan R.G., Guan X.H., Feng Z.X., Chen H., Chen Y. (2014). Microstructures and Properties of Graphene-Cu/Al Composite Prepared by a Novel Process Through Clad Forming and Improving Wettability with Copper. Adv. Eng. Mater..

[28] Katin K.P., Kochaev A.I., Bereznitskiy I.V., Kalika E.B., Kaya S., Flores-Moreno R., Maslov M.M. (2023). Interaction of pristine and novel graphene allotropes with copper nanoparticles: Coupled density functional and molecular dynamics study. Diam. Relat. Mater..

[29] Garzon A., Wang S., Omoniyi A., Tam L., Che F., Hensley A.J. (2024). Temperature and pressure driven functionalization of graphene with hydrogen and oxygen via ab initio phase diagrams. Appl. Surf. Sci..

[30] Barkov P.V., Slepchenkov M.M., Glukhova O.E. (2024). Current flow patterns in graphene nanomesh films functionalized with carbonyl and carboxyl groups. Lett. Mater..

[31] Biji C.A., Janardhanan J.C., John H. (2024). Facile interface engineering of highly stable covalently functionalized graphene oxide-nickel based metal organic framework heterostructures for supercapacitor applications. Electrochim. Acta.

[32] Wang S., Guo X., Li K., Wang G., Su S., Wu J., Li L., Xie Y., Guo C., Pan K. (2023). Monodispersed Ni12P5 nanocrystals in situ grown on reduced graphene oxide matrix with enhanced Li-electrochemical properties. J. Alloys Compd..

[33] Rodrigues D.C., Amorim R.G., Latgé A., Venezuela P. (2023). Improving the sensitivity of graphyne nanosensor by transition metal doping. Carbon.

[34] Holec D., Kostoglou N., Tampaxis C., Babic B., Mitterer C., Rebholz C. (2018). Theory-guided metal-decoration of nanoporous carbon for hydrogen storage applications. Surf. Coatings Technol..

[35] Guan R., Wang Y., Zheng S., Su N., Ji Z., Liu Z., An Y., Chen B. (2019). Fabrication of aluminum matrix composites reinforced with Ni-coated graphene nanosheets. Mater. Sci. Eng. A.

[36] Luo Y., Huang Y., Hassan A., Quanfang C. (2022). Nickel-encapsulated graphene reinforced aluminum matrix composites with increased mechanical strength and electrical conductivity. J. Mater. Res..

[37] Liu G., Zhao N., Shi C., Liu E., He F., Ma L., Li Q., Li J., He C. (2017). In-situ synthesis of graphene decorated with nickel nanoparticles for fabricating reinforced 6061Al matrix composites. Mater. Sci. Eng. A.

[38] Soni K., Panwar N.L., Lanjekar P.R. (2024). Emergence of carbonaceous material for hydrogen storage: An overview. Clean Energy.

[39] Wang X., Xiao W., Wang J., Sun L., Shi J., Guo H., Liu Y., Wang L. (2021). Enhanced interfacial strength of graphene reinforced aluminum composites via X (Cu, Ni, Ti)-coating: Molecular-dynamics insights. Adv. Powder Technol..

[40] Han R., Song H., Wang J., Li Y. (2021). Strengthening mechanism of Al matrix composites reinforced by nickel-coated graphene: Insights from molecular dynamics simulation. Phys. B Condens. Matter.

[41] Zhang S., Yang N., Tang Y., Ma C., Peng H., Chang G., Li L., Li X., Zhang W., Elmarakbi A. (2024). Achieving better strength-toughness synergy in heterogeneous Cu/Ni/graphene composites: A molecular dynamics simulation. Mater. Today Commun..

[42] Yang Y., Liu M., Zhou S., Ren W., Zhou Q., Zhang W. (2021). Strengthening behaviour of continuous graphene network in metal matrix composites. Carbon.

[43] Safina L.R., Krylova K.A., Baimova J.A. (2022). Molecular dynamics study of the mechanical properties and deformation behavior of graphene/metal composites. Mater. Today Phys..

[44] Vardanyan V.H., Urbassek H.M. (2021). Morphology of graphene flakes in Ni-graphene nanocomposites and its influence on hardness: An atomistic study. Carbon.

[45] Yang Y., Liu M., Du J., Zhang W., Zhou S., Ren W., Zhou Q., Shi L. (2022). Construction of graphene network in Ni matrix composites: A molecular dynamics study of densification process. Carbon.

[46] Wu P., Wei T., Wei J., Zhou Q., Zhang W., Liu M. (2024). Simulation on fabricating graphene-coated nickel powders through micromechanical exfoliation. Mater. Today Commun..

[47] Safina L.R., Rozhnova E.A., Krylova K.A., Murzaev R.T., Baimova J.A. (2024). Interatomic potentials for graphene reinforced metal composites: Optimal choice. Comput. Phys. Commun..

[48] Wu Y., An C., Guo Y., Zong Y., Jiang N., Zheng Q., Yu Z.Z. (2024). Highly Aligned Graphene Aerogels for Multifunctional Composites. Nano-Micro Lett..

[49] Zhang F., Liu J., Hu L., Guo C. (2024). Recent Progress of Three-Dimensional Graphene-Based Composites for Photocatalysis. Gels.

[50] Cao L., Wang C., Huang Y. (2023). Structure optimization of graphene aerogel-based composites and applications in batteries and supercapacitors. Chem. Eng. J..

[51] Qiu C., Su Y., Yang J., Chen B., Ouyang Q., Zhang D. (2021). Structural modelling and mechanical behaviors of graphene/carbon nanotubes reinforced metal matrix composites via atomic-scale simulations: A review. Compos. Part C Open Access.

[52] Safina L., Baimova J., Krylova K., Murzaev R., Mulyukov R. (2020). Simulation of metal-graphene composites by molecular dynamics: A review. Lett. Mater..

[53] Yazdani M., Ghassemi A., Shahgholi M., Fesharaki J.J., Galehdari S.A. (2024). Molecular dynamics method to investigate the interaction energy and mechanical properties of the reinforced graphene aerogel with paraffin as the phase change material in the presence of different external heat fluxes. J. Taiwan Inst. Chem. Eng..

[54] Wang Q., Wang L., Wu Y. (2024). Study on tensile properties of copper/graphene/2D-SiC composite materials: A molecular dynamics simulation. Mater. Today Commun..

[55] Krylova K.A., Safina L.R., Murzaev R.T., Baimova J.A., Mulyukov R.R. (2021). Effect of Nanoparticle Size on the Mechanical Strength of Ni–Graphene Composites. Materials.

[56] Murzaev R.T., Krylova K.A., Baimova J.A. (2023). Thermal Expansion and Thermal Conductivity of Ni/Graphene Composite: Molecular Dynamics Simulation. Materials.

[57] Sarkar K., Talukder M.A. (2024). Structurally realistic carbide-derived carbon model in annealing molecular dynamics methodology with analytic bond-order potential. Mater. Adv..

[58] Stuart S.J., Tutein A.B., Harrison J.A. (2000). A reactive potential for hydrocarbons with intermolecular interactions. J. Chem. Phys..

[59] Orekhov N., Ostroumova G., Stegailov V. (2020). High temperature pure carbon nanoparticle formation: Validation of AIREBO and ReaxFF reactive molecular dynamics. Carbon.

[60] Rozhkov M., Abramenko N., Kolesnikova A., Romanov A. (2020). Zero misorientation interfaces in graphene. Lett. Mater..

[61] Zhou J., Shen J., Yue W., Liu Y., Chen Z. (2023). Molecular dynamics simulation of reinforcement mechanism of graphene/aluminum composites and microstructure evolution. J. Mater. Res. Technol..

[62] Katin K.P., Prudkovskiy V.S., Maslov M.M. (2018). Molecular dynamics simulation of nickel-coated graphene bending. Micro Nano Lett..

[63] Galashev A.Y., Katin K.P., Maslov M.M. (2019). Morse parameters for the interaction of metals with graphene and silicene. Phys. Lett. A.

[64] Katin K., Kaya S., Maslov M. (2022). Graphene nanoflakes and fullerenes doped with aluminum: Features of Al-C interaction and adsorption characteristics of carbon shell. Lett. Mater..

[65] Huang S.P., Mainardi D.S., Balbuena P.B. (2003). Structure and dynamics of graphite-supported bimetallic nanoclusters. Surf. Sci..

[66] Tavenner J.P., Mendelev M.I., Neuberger R., Arroyave R., Otis R., Lawson J.W. (2024). Determination of *γ*/*γ*’ interface free energy for solid state precipitation in Ni–Al alloys from molecular dynamics simulation. J. Chem. Phys..

[67] Fischer F., Schmitz G., Eich S. (2019). A systematic study of grain boundary segregation and grain boundary formation energy using a new copper–nickel embedded-atom potential. Acta Mater..

[68] Somlyai-Sipos L., Janovszky D., Sycheva A., Baumli P. (2020). Investigation of the Melting Point Depression of Copper Nanoparticles. IOP Conf. Ser. Mater. Sci. Eng..

[69] Puri P., Yang V. (2007). Effect of Particle Size on Melting of Aluminum at Nano Scales. J. Phys. Chem. C.

[70] LAMMPS Large-Scale Atomic/Molecular Massively Parallel Simulator. https://www.lammps.org/.

[71] Plimpton S. (1995). Fast Parallel Algorithms for Short-Range Molecular Dynamics. J. Comput. Phys..

[72] Thompson A.P., Aktulga H.M., Berger R., Bolintineanu D.S., Brown W.M., Crozier P.S., in ’t Veld P.J., Kohlmeyer A., Moore S.G., Nguyen T.D. (2022). LAMMPS—A flexible simulation tool for particle-based materials modeling at the atomic, meso, and continuum scales. Comput. Phys. Commun..

[73] VMD Molecular Graphics Viewer. https://www.ks.uiuc.edu/Research/vmd/.

[74] OVITO Open Visualization Tool. https://www.ovito.org/.

